# Prognostic Significance of Albumin–Bilirubin (ALBI) Score in Gastric Cancer Patients Undergoing Curative Resection Followed by Adjuvant Therapy

**DOI:** 10.3390/medicina62020337

**Published:** 2026-02-07

**Authors:** Talat Aykut, Oğuzhan Yıldız, Bahattin Engin Kaya, Ali Fuat Gürbüz, Mustafa Korkmaz, Mehmet Zahid Koçak, Melek Karakurt Eryılmaz, Murat Araz, Mehmet Artaç

**Affiliations:** 1 Department of Medical Oncology, Necmettin Erbakan University School of Medicine, Konya 42080, Turkey; dr.oguzhan@outlook.com (O.Y.); md.enginkaya@gmail.com (B.E.K.); dr.alifuatgurbuz@hotmail.com (A.F.G.); mehmetzahidkocak@hotmail.com (M.Z.K.); drangelkarakurt@hotmail.com (M.K.E.); zaratarum@yahoo.com (M.A.); mehmetartac@yahoo.com (M.A.); 2 Department of Medical Oncology, Suleyman Demirel University School of Medicine, Isparta 32260, Turkey; dr.musstafa@gmail.com

**Keywords:** Albumin–Bilirubin (ALBI) score, gastric cancer, prognosis, overall survival, recurrence-free survival

## Abstract

*Background and Objectives*: Gastric cancer is an aggressive malignancy characterized by high recurrence rates, even following curative resection. The Albumin–Bilirubin (ALBI) score was originally established to assess hepatic functional reserve in patients with hepatocellular carcinoma (HCC). By reflecting both systemic inflammation and nutritional status, the ALBI score has demonstrated significant prognostic utility across a spectrum of solid malignancies. The present study aimed to evaluate the prognostic significance of the ALBI score in gastric cancer patients receiving adjuvant therapy after curative-intent resection. *Materials and Methods*: This retrospective study included 168 patients with gastric cancer who underwent curative-intent resection followed by adjuvant therapy between November 2008 and January 2021. ALBI scores were calculated from pre-treatment serum albumin and bilirubin levels. Patients were dichotomized into ALBI Grade 1 and ALBI Grade 2 based on an optimal ROC-derived cut-off value of −2.60. Survival outcomes, including overall survival (OS) and recurrence-free survival (RFS), were estimated using the Kaplan–Meier method and compared via the log-rank test. Independent prognostic factors were identified using univariate and multivariate Cox proportional hazards regression models. *Results*: Of the 168 patients, 56.5% were classified as ALBI Grade 1 and 43.5% as ALBI Grade 2. ALBI Grade 2 was associated with significantly shorter median RFS (18.7 vs. 72.2 months; *p* = 0.001) and OS (40.7 vs. 104.3 months; *p* = 0.003). Multivariable analysis identified ALBI Grade 2 as an independent predictor for both poor OS (HR: 1.699, *p* = 0.010) and RFS (HR: 1.767, *p* = 0.004). Pathological stage III disease was also a significant independent prognostic factor for OS (HR: 3.024) and RFS (HR: 3.049) (all *p* = 0.010). Additionally, elevated CEA correlated with shorter RFS (*p* = 0.023). *Conclusions*: The ALBI score is a prognostic marker for both overall and recurrence-free survival in gastric cancer patients receiving adjuvant therapy. A lower ALBI score is associated with longer survival outcomes. The ALBI score may support postoperative risk stratification and individualized follow-up planning.

## 1. Introduction

Gastric cancer is the fifth most commonly diagnosed cancer worldwide and the fourth leading cause of cancer-related death [1]. Only about 30% of patients present with localized disease at the time of diagnosis [2]. Curative resection remains the therapeutic mainstay for localized gastric cancer. However, due to the tumor’s markedly aggressive biological profile, the clinical course following curative surgery often remains difficult to predict. A substantial proportion of patients experience early postoperative recurrence, with distant metastatic recurrences in particular significantly compromising survival outcomes [3]

The high risk of recurrence in these patients necessitates closer postoperative surveillance. During follow-up, physical examination, blood tests, and radiological imaging are routinely employed. However, frequent radiological imaging is not cost-effective. In the postoperative setting, identifying reliable, cost-effective, and easily accessible biomarkers that can predict prognosis is of significant clinical importance.

Albumin is a protein that reflects hepatic synthetic capacity, nutritional status, and systemic inflammation; low levels indicate both impaired liver reserve and heightened inflammatory activity [4,5]. Bilirubin is a sensitive marker of hepatocellular clearance and bile flow; it is also strongly influenced by hepatocellular injury, cholestasis, oxidative stress, and inflammatory processes. Elevated bilirubin levels have been shown in various studies to be closely associated with macrophage activation, cytokine response, and oxidative stress burden [6,7]. The Albumin–Bilirubin (ALBI) score, developed based on the prognostic relevance of these two biochemical parameters, was originally designed to assess hepatic functional reserve in patients with hepatocellular carcinoma (HCC) [8]. Designed as an alternative to the Child–Pugh classification, it has emerged as a simpler and more objective model, owing to the exclusion of subjective parameters such as ascites and encephalopathy. Over time, the ALBI score has been recognized not only as a reflection of liver function but also as an indicator of systemic inflammation and nutritional status, leading to its adoption as a prognostic marker in various solid tumors [9].

Studies conducted in various gastrointestinal malignancies, including colorectal, pancreatic, gastric, and esophageal cancers, have demonstrated that the ALBI score serves as a valuable prognostic biomarker and may influence tumor biology and treatment response. The consistent finding across these studies is that higher ALBI scores are associated with poorer prognosis [10,11,12,13]. Similarly, evidence also supports the ALBI score as an independent prognostic factor in non-gastrointestinal malignancies, such as lung cancer [14,15]. However, data on the prognostic role of the ALBI score in gastric cancer patients receiving adjuvant therapy following curative resection remain limited.

In the postoperative period, patient prognosis is determined not only by tumor-related factors but also by host-related physiological parameters. Given the pronounced systemic stress response following surgery and the potential hepatotoxicity of adjuvant therapies, the ALBI score is hypothesized to be a biologically meaningful prognostic marker in gastric cancer patients [16]. Accordingly, evaluating the impact of the ALBI score on survival outcomes in gastric cancer patients receiving adjuvant therapy after curative resection emerges as an important area of research—both for personalizing clinical management and for the early identification of high-risk patients.

The aim of this study was to evaluate the prognostic value of the ALBI score for overall survival (OS) and recurrence-free survival (RFS) in gastric cancer patients receiving adjuvant therapy following curative resection. This study distinguishes itself from previous literature by evaluating the prognostic value of the ALBI score in a homogeneous cohort of gastric cancer patients who exclusively underwent curative resection followed by adjuvant therapy.

## 2. Materials and Methods

**Figure 1:**
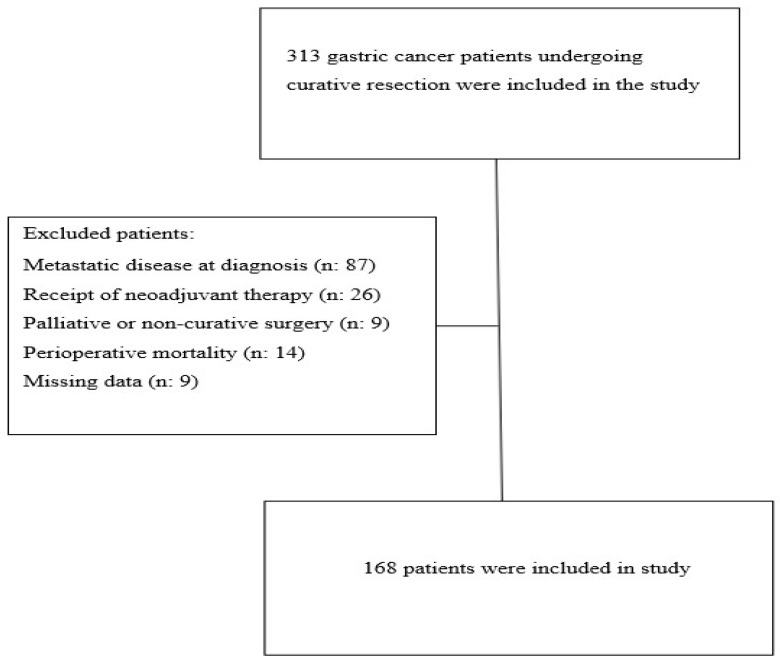
Flow chart of the study.

This retrospective cohort study included 168 patients with gastric cancer who underwent curative resection followed by adjuvant chemotherapy at our institution between November 2008 and January 2021. Inclusion criteria were defined as having a histologically confirmed diagnosis of gastric adenocarcinoma, undergoing curative-intent surgical resection followed by adjuvant chemotherapy, and having complete and regularly documented clinical and laboratory records in the institutional database, including baseline serum albumin and total bilirubin levels required for ALBI score calculation. Exclusion criteria included the presence of metastatic disease at diagnosis, receipt of neoadjuvant therapy, palliative or non-curative surgery, perioperative mortality, and missing laboratory data precluding the calculation of the ALBI score. A total of 313 gastric cancer patients who underwent curative-intent resection during the study period were screened. Of these, 145 were excluded due to metastatic disease at diagnosis (n = 87), receipt of neoadjuvant therapy (n = 26), palliative or non-curative surgery (n = 9), perioperative mortality (n = 14), or missing data (n = 9). The remaining 168 patients constituted the final study cohort and were included in the analysis (Figure 1).

Adjuvant chemotherapy regimens consisted of fluoropyrimidine-based and/or platinum-based combinations administered according to institutional practice and contemporary guidelines. The prognostic impact of the ALBI score was evaluated independently of specific chemotherapy regimens. Demographic characteristics (age, sex) and clinicopathological variables, including surgical stage, type of dissection and resection, tumor grade, lymphovascular invasion (LVI), and perineural invasion (PNI), were retrospectively retrieved from medical records.

Patients with clinically documented chronic liver disease requiring specific treatment were not routinely encountered in the study cohort. Detailed etiologic classification of liver disease and postoperative complication grading were not uniformly available and therefore not included in the analysis.

The ALBI score was calculated for all patients using serum albumin (g/dL) and total bilirubin (mg/dL) levels obtained at the time of diagnosis, according to the following formula: ALBI = (log_10_ bilirubin [mg/dL] × 0.66) + (albumin [g/dL] × −0.085). The optimal cut-off value specific to the study population was determined using Receiver Operating Characteristic (ROC) curve analysis and was identified as −2.60. This cut-off was internally derived in the current cohort and should be considered exploratory. Based on the calculated ALBI scores, patients were divided into two groups: ALBI Grade 1 (≤−2.60) and ALBI Grade 2 (>−2.60).

### Statistical Analysis

The primary endpoints of the study were OS and RFS. OS was defined as the time from diagnosis to death from any cause, while RFS was defined as the time from surgery to either recurrence or death. Survival analyses were performed using the Kaplan–Meier method, and differences between groups were evaluated with the log-rank test. Categorical variables were compared using the Pearson chi-square test. Prognostic factors were analyzed using univariate and multivariate Cox proportional hazards regression models. Missing data were handled using a complete-case approach. Patients were stratified by treatment era (2008–2016 vs. 2017–2021) to account for secular changes in perioperative care and adjuvant treatment standards, and treatment era was included as a covariate in Cox models. Variables with a *p*-value < 0.05 in univariate analysis, along with clinically relevant factors, were included in the multivariate Cox regression model. The proportional hazards assumption was formally evaluated using time-dependent covariate analysis. Evidence of non-proportionality was observed for ALBI. Therefore, stratified Cox proportional hazards models were applied, stratifying by disease stage (stage I–II vs. stage III), to allow for non-proportional baseline hazards across strata. Analyses were conducted using a complete-case approach, and hazard ratios are reported with 95% confidence intervals. All statistical analyses were performed using IBM SPSS Statistics version 22.0 (version 22.0, SPSS Inc., Chicago, IL, USA), and a *p*-value of <0.05 was considered statistically significant.

## 3. Results

Among 313 screened patients, 145 were excluded according to the predefined exclusion criteria, and 168 patients were included in the final analysis (Figure 1). Of these, 95 patients (56.5%) were classified as ALBI Grade 1 and 73 (43.5%) as ALBI Grade 2. The median age of the cohort was 59 years (range, 29–84); 113 patients (67.3%) were <65 years old and 55 (32.7%) were ≥65 years old. The proportion of patients aged ≥ 65 years was significantly higher in the ALBI Grade 2 group compared with the ALBI Grade 1 group (42.5% vs. 25.3%, *p* = 0.014). Male predominance was also greater in the ALBI Grade 2 group (76.7% vs. 60.0%, *p* = 0.016). No statistically significant differences were observed between the groups regarding smoking status (*p* = 0.255), type of lymph node dissection (*p* = 0.274), resection status (*p* = 0.126), postoperative pathological stage (*p* = 0.843), tumor grade (*p* = 0.872), lymphovascular invasion (*p* = 0.395), perineural invasion (*p* = 0.118), BMI categories (*p* = 0.524), neutrophil-to-lymphocyte ratio (*p* = 0.559), or ECOG performance status (*p* = 0.146), indicating comparable baseline clinicopathologic characteristics between the ALBI Grade 1 and ALBI Grade 2 groups (Table 1).

Baseline liver function parameters were systematically evaluated using serum AST, ALT and INR levels. Median AST values were 31.3 (IQR 10) in ALBI Grade 1 and 25.0 (IQR 5) in ALBI Grade 2 patients. Median ALT values were 28.0 (IQR 16) and 24.0 (IQR 17), respectively. Median INR levels were within normal limits in both groups (1.02 (IQR 0.07) vs. 1.01 (IQR 0.06)). No statistically significant or clinically meaningful differences in baseline liver function parameters were observed between the ALBI groups.

The median follow-up duration was 94.4 months (95% CI, 85.8–103.0), estimated using the reverse Kaplan–Meier method. During follow-up, a total of 102 deaths occurred (ALBI Grade 1: n = 47; ALBI Grade 2: n = 55), and 109 recurrence events were observed overall (ALBI Grade 1: n = 51; ALBI Grade 2: n = 58).

To account for potential secular changes over the long study period, patients were stratified into two treatment eras according to the year of diagnosis (2008–2016 vs. 2017–2021). The median OS was 54.8 months (95% CI, 34.3–75.2) in the 2008–2016 period and 46.1 months (95% CI, 21.2–71.1) in the 2017–2021 period, with no statistically significant difference between eras (log-rank *p* = 0.945). Similarly, median RFS was 29.0 months (95% CI, 11.3–46.8) in the 2008–2016 period and 41.2 months (95% CI, 15.4–67.1) in the 2017–2021 period, with no significant difference between eras (log-rank *p* = 0.800).

In era-specific analyses, patients diagnosed between 2008 and 2016 showed a numerically longer median OS in the ALBI Grade 1 group compared to the ALBI Grade 2 group (96.8 vs. 43.9 months), although this did not reach statistical significance (log-rank *p* = 0.062). In contrast, among patients diagnosed between 2017 and 2021, median OS was not reached in the ALBI Grade 1 group, whereas it was 23.5 months (95% CI, 1.0–46.0) in the ALBI Grade 2 group, demonstrating a significant survival difference across ALBI categories (log-rank *p* = 0.014). Regarding RFS, ALBI Grade 1 was associated with significantly longer survival compared to ALBI Grade 2 in both eras (2008–2016: 50.7 months, 95% CI 0.0–183.4 vs. 25.6 months, 95% CI 6.6–44.5; log-rank *p* = 0.015; 2017–2021: 66.0 months, 95% CI 0.0–138.4 vs. 14.3 months, 95% CI 6.9–21.8; log-rank *p* = 0.036).

Chemotherapy dose reduction was observed in 46 patients (27.4%), whereas 122 patients (72.6%) completed treatment without dose reduction. When stratified by ALBI Grade, dose reduction occurred in 28 of 95 patients (29.5%) with ALBI Grade 1 and in 18 of 73 patients (24.7%) with ALBI Grade 2. There was no statistically significant association between ALBI Grade and chemotherapy dose reduction (χ^2^ = 0.48, *p* = 0.488). These findings indicate that chemotherapy dose reduction rates were comparable between ALBI Grade groups.

Adjuvant chemotherapy regimens differed significantly between ALBI grade groups. Overall, 102 patients (60.7%) received FOLFOX/XELOX, 35 patients (20.8%) received FUFA, and 31 patients (18.5%) received CF. Among patients with ALBI Grade 1, FOLFOX/XELOX was administered in 61 patients (64.2%), FUFA in 12 patients (12.6%), and CF in 22 patients (23.2%). In contrast, among patients with ALBI Grade 2, FOLFOX/XELOX was used in 41 patients (56.2%), FUFA in 23 patients (31.5%), and CF in 9 patients (12.3%). The distribution of adjuvant chemotherapy regimens differed significantly according to ALBI Grade (*p* = 0.006).

**Figure 2:**
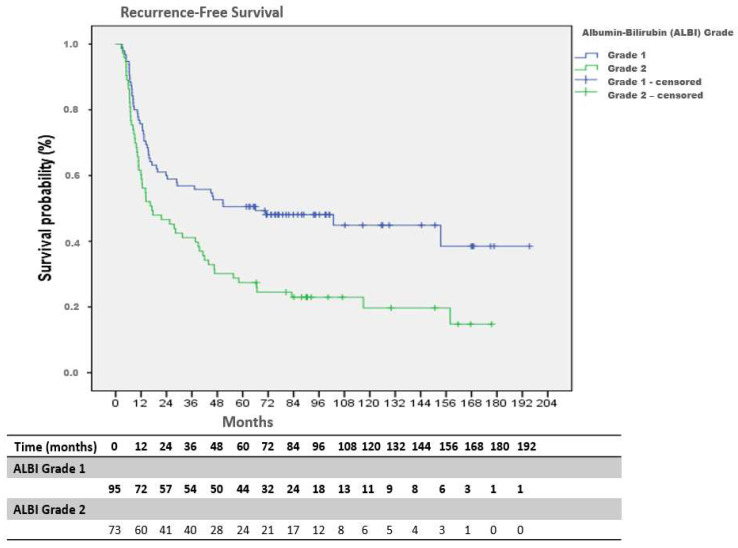
Kaplan–Meier curves for recurrence-free survival (RFS) stratified by ALBI grade, with numbers at risk.

There was no significant association between ALBI grade and pathological stage (Stage III: 56.8% in Grade 1 vs. 56.2% in Grade 2; *p* = 0.843) or resection status (R0: 67.4% vs. 63.0%; *p* = 0.126). Median RFS was 72.2 months in the ALBI Grade 1 group and 18.7 months in the ALBI Grade 2 group. This difference was statistically significant (log-rank *p* = 0.001) (Figure 2).

**Figure 3:**
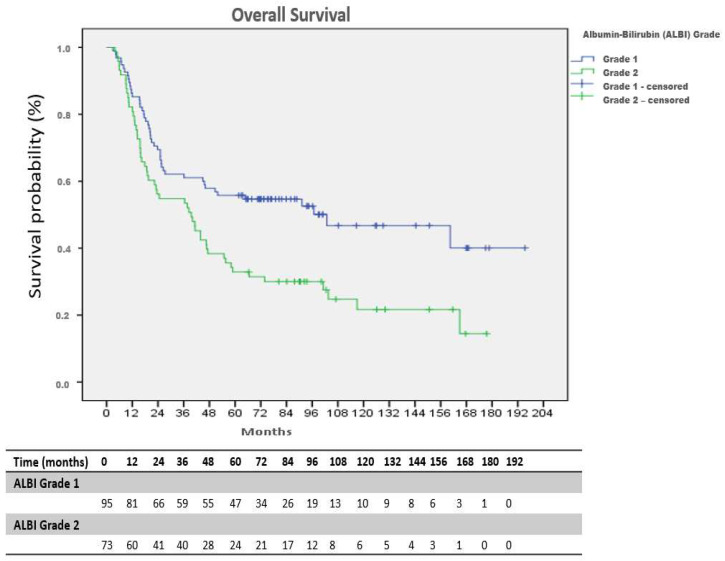
Kaplan–Meier curves for overall survival (OS) stratified by ALBI grade, with numbers at risk.

Median OS was 104.3 months in the ALBI Grade 1 group and 40.7 months in the Grade 2 group, demonstrating a significant survival advantage in favor of the lower ALBI group (log-rank *p* = 0.003) (Figure 3).

The estimated 1-, 3-, and 5-year overall survival rates in the ALBI Grade 1 group were 69% (95% CI: 59–79), 58% (95% CI: 48–68), and 46% (95% CI: 34–58), respectively. Corresponding survival rates in the ALBI Grade 2 group were 56% (95% CI: 44–68), 38% (95% CI: 26–50), and 20% (95% CI: 10–30). Overall survival was significantly longer in patients with ALBI Grade 1 compared with Grade 2 (log-rank *p* = 0.002) (Table 2). The estimated 1-, 3-, and 5-year recurrence-free survival rates in the ALBI Grade 1 group were 60% (95% CI: 50–70), 53% (95% CI: 43–63), and 43% (95% CI: 31–55), respectively, compared with 47% (95% CI: 35–59), 30% (95% CI: 20–40), and 16% (95% CI: 6–26) in the ALBI Grade 2 group (Table 2).

In the univariable Cox regression analysis, ALBI Grade 2 was significantly associated with shorter OS (HR: 1.797; 95% CI: 1.217–2.653; *p* = 0.003). Elevated CEA levels were also associated with poorer OS (HR: 1.720; 95% CI: 1.169–2.567; *p* = 0.006), and patients aged ≥ 65 years had worse survival outcomes (HR: 1.592; 95% CI: 1.071–2.367; *p* = 0.021). Additionally, postoperative stage III disease was significantly associated with poorer OS (HR: 3.064; 95% CI: 1.994–4.709; *p* = 0.001). In contrast, the extent of lymphadenectomy and resection status were not significant predictors (*p* > 0.05 for both).

In the multivariable Cox proportional hazards regression model, ALBI Grade 2 (HR: 1.699; 95% CI: 1.132–2.550; *p* = 0.010) and postoperative stage III disease (HR: 3.024; 95% CI: 1.960–4.666; *p* = 0.010) remained independently associated with OS, whereas age and carcinoembryonic antigen (CEA) levels lost statistical significance after adjustment (Table 3). The base model including postoperative stage alone yielded a C-index of 0.70, which increased to 0.75 after inclusion of ALBI, indicating incremental prognostic value.

To further explore whether the prognostic effect of ALBI was driven solely by pathological stage, stage-stratified Cox proportional hazards analyses were performed. Among patients with stage I–II disease, higher ALBI scores were significantly associated with worse overall survival (HR per 1-point increase: 2.68; *p* = 0.010). Similarly, in patients with stage III disease, higher ALBI scores remained significantly associated with increased mortality risk (HR per 1-point increase: 1.70; *p* = 0.026). In addition, in stratified Cox proportional hazards models stratified by pathological stage, higher ALBI scores remained significantly associated with worse overall survival (HR per 1-point increase: 1.93; *p* = 0.001).

In the univariable Cox regression analysis for RFS, ALBI Grade 2 was significantly associated with shorter RFS (HR: 1.850; 95% CI: 1.269–2.698; *p* = 0.001). Elevated CEA levels (HR: 1.878; 95% CI: 1.284–2.747; *p* = 0.001), age ≥ 65 years (HR: 1.477; 95% CI: 1.003–2.174; *p* = 0.048), and postoperative stage III disease (HR: 3.116; 95% CI: 2.061–4.711; *p* = 0.001) were also significantly associated with poorer RFS. In contrast, the extent of lymphadenectomy and resection status were not significant predictors (*p* > 0.05 for both).

In the multivariable Cox proportional hazards regression model, ALBI Grade 2 (HR: 1.767; 95% CI: 1.011–4.622; *p* = 0.004), elevated CEA levels (HR: 1.591; 95% CI: 1.067–2.373; *p* = 0.023), and postoperative stage III disease (HR: 3.049; 95% CI: 2.011–4.622; *p* = 0.010) remained independent prognostic factors for RFS, whereas age lost statistical significance after adjustment (Table 4).

When modeled as a continuous variable, ALBI remained significantly associated with OS and RFS, indicating that the prognostic effect was not dependent on arbitrary categorization. To explore whether the prognostic association observed with ALBI might be driven predominantly by its individual components, survival outcomes were additionally evaluated according to baseline serum albumin (low vs. normal) and total bilirubin (≤0.4550 vs. >0.4550 mg/dL) categories. Patients with low baseline albumin had a numerically shorter median RFS compared to those with normal albumin levels (27.6 months, 95% CI 5.3–49.8 vs. 46.1 months, 95% CI 6.3–86.0), although this difference did not reach statistical significance (log-rank *p* = 0.081). Similarly, median OS was 43.9 months (95% CI 29.0–58.8) in the low-albumin group and 55.7 months (95% CI 1.3–110.0) in the normal-albumin group (log-rank *p* = 0.203). For total bilirubin, an ROC curve analysis identified 0.4550 mg/dL as the optimal cut-off value for OS prediction (sensitivity 55%, specificity 53%). Using this threshold, no significant differences were observed in OS (45.0 vs. 51.9 months; log-rank *p* = 0.731) or RFS (28.8 vs. 39.0 months; log-rank *p* = 0.972).

## 4. Discussion

In this study, the ALBI score was independently associated with both RFS and OS in gastric cancer patients who received adjuvant therapy following curative resection. The finding that lower ALBI scores are associated with longer survival supports the clinical relevance of this parameter as a biomarker that reflects not only hepatic function but also the host’s inflammatory and nutritional reserve. This study underscores the need for innovative and easily applicable models to improve prognostic assessment in the adjuvant setting and highlights the ALBI score as a promising candidate in this context.

In the gastric cancer literature, there is a growing body of evidence linking preoperative or early perioperative ALBI scores with both short- and long-term outcomes. These findings, which demonstrate that the ALBI score reflects not only hepatic reserve but also systemic inflammation and the host’s metabolic status, have shown that higher ALBI scores are associated with increased postoperative complications and reduced survival. Our study makes a significant contribution to the existing literature by evaluating the impact of the ALBI score on both recurrence-free and overall survival in a homogeneous cohort of patients who underwent curative resection followed by adjuvant therapy. These findings suggest that the ALBI score may serve as a useful tool for postoperative risk stratification in gastric cancer patients receiving adjuvant treatment.

Zhu et al. reported that the preoperative ALBI score predicted both short- and long-term outcomes in patients with locally advanced gastric cancer; a higher ALBI score was associated with increased complication rates and shorter survival, particularly in stage II–III patients [17]. Consistent with the literature, Kanda et al. reported that a higher preoperative ALBI score was associated with poorer survival following curative gastrectomy in patients with pT2–T4 gastric cancer [18]. Similarly, Wang et al. demonstrated that the ALBI score served as an independent prognostic indicator in stage I–III gastric cancer, regardless of TNM stage [19].

The prognostic value of the ALBI score has been demonstrated not only in gastric cancer but also across other gastrointestinal malignancies. In a systematic review and meta-analysis conducted by Xu et al., higher ALBI scores were significantly associated with poorer overall survival in patients with HCC [20]. Furthermore, Lee et al. demonstrated that the ALBI score served as an independent prognostic factor for predicting early recurrence following curative resection in HCC [21]. In the literature, a systematic review by Peng et al. showed that the ALBI score had superior discriminatory power compared to the Child–Pugh classification in predicting prognosis in patients with HCC [22]. Additionally, Demircan et al. confirmed that a higher ALBI score was associated with poorer survival in patients with colorectal cancer with liver metastases [10]. Mejia et al. reported that a higher ALBI score was associated with increased mortality in patients with pancreatic ductal adenocarcinoma who underwent Whipple surgery [23]. All of these findings indicate suggest that the ALBI score is a host-based prognostic biomarker across gastrointestinal malignancies. Our findings support the notion that the ALBI score may serve as a reliable and reproducible prognostic biomarker, with potential clinical utility in the management of gastric cancer. Similar findings have also been reported in non-oncological settings. For instance, Kawata et al. demonstrated that elevated ALBI scores were associated with increased in-hospital mortality among patients with acute heart failure [24].

In our study, the lack of association between the ALBI score and pathological stage or type of resection suggests that ALBI may reflect host-related biological processes rather than tumor burden itself. Although albumin and bilirubin may be influenced by liver disease, inflammatory conditions, and postoperative complications, the absence of significant differences in BMI, ECOG performance status, NLR, pathological stage, and resection status between ALBI groups suggests that the prognostic impact of ALBI was not merely driven by overt hepatic dysfunction or postoperative morbidity. The relatively high rate of R1 resections in our cohort likely reflects the inclusion of patients with locally advanced tumors, in whom achieving microscopically negative margins may be challenging despite curative-intent surgery.

In this context, ALBI appears to reflect a broader host-related vulnerability rather than isolated liver dysfunction. Accordingly, ALBI should be interpreted as a prognostic indicator rather than a causal determinant of oncologic outcomes. Although age and CEA levels were associated with survival in univariate analyses, only the ALBI score and stage III disease remained independent prognostic factors in the multivariate analysis. This finding suggests that the ALBI score provides additional and independent prognostic information beyond conventional clinicopathological factors.

ALBI Grade 2 may reflect aggressive disease biology, occult metastatic burden, systemic inflammation, or biliary/hepatic involvement rather than a direct causal pathway from baseline liver function to oncologic outcome. To explore whether the observed prognostic effect was driven by the composite score rather than its individual components, we evaluated albumin and bilirubin separately. Neither albumin nor bilirubin alone demonstrated a significant association with survival outcomes, whereas the composite ALBI score remained independently prognostic. This finding suggests that ALBI captures integrated biological information beyond isolated laboratory parameters and does not merely reflect a single dominant component. Nevertheless, the potential for residual confounding by disease burden cannot be fully excluded, and the results should be interpreted in a prognostic rather than causal framework. Importantly, despite demographic differences between ALBI groups, the absence of significant differences in postoperative stage, key pathological risk factors, BMI, ECOG performance status and NLR suggests that the prognostic impact of ALBI was not solely driven by advanced disease burden or overt nutritional and inflammatory status

The demonstrated prognostic value of the ALBI score across various tumor types is consistent with its biological underpinnings. Albumin serves as a marker sensitive to systemic inflammation and catabolic processes, with decreased levels being associated with immunosuppression, malnutrition, and an increased risk of tumor progression. Total bilirubin, by reflecting mechanisms of oxidative stress and immune regulation, provides insight into the host’s overall physiological capacity. These insights offer a biologically plausible explanation for the observed association between higher ALBI scores and shorter RFS and OS. In addition, hepatic function was also evaluated using baseline AST, ALT, and INR levels, which generally suggested preserved liver function across the cohort.

One notable strength of this study is the inclusion of a homogeneous patient population who underwent curative surgery and received adjuvant therapy. This allowed for the evaluation of the prognostic impact of the ALBI score independent of treatment-related heterogeneity. This study also has several limitations. First, the retrospective and single-center design may increase the risk of selection bias. Second, calculating the ALBI score based on baseline laboratory values may limit the ability to fully account for perioperative biochemical variability or coexisting cholestatic conditions. Although ECOG performance status was available and included in the analyses, a formal comorbidity burden assessment using standardized indices such as the Charlson Comorbidity Index could not be performed due to the retrospective nature of the study and incomplete documentation of comorbid conditions. Third, detailed chemotherapy delivery parameters (e.g., cycle numbers, cumulative dose intensity, and toxicity profiles) were not uniformly available and could not be comprehensively included in the analysis. Additionally, because the ALBI Grade 1/2 cut-off was internally derived in the current cohort, it should be considered exploratory and may be unstable across different populations. Further validation in independent cohorts is warranted to confirm the generalizability of these findings. A formal comparison between included and excluded patients could not be performed due to incomplete baseline clinical and laboratory data among excluded cases. A further limitation is that advanced performance metrics such as calibration and reclassification analyses (e.g., NRI) were not evaluated due to the retrospective single-center design, limited sample size, and absence of an external validation cohort.

The clinical implication of our study is that the ALBI score may serve as a practical tool for risk stratification in the adjuvant setting. In patients with high ALBI scores, more intensive follow-up strategies, early nutritional support, and prioritization of multimodal rehabilitation approaches may be considered. This approach may support daily clinical practice by providing a more realistic framework for identifying which patients require closer monitoring in the post-adjuvant period. These implications should be interpreted as hypothesis-generating and require prospective validation.

## 5. Conclusions

This study demonstrates that the ALBI score is an independent prognostic indicator for both RFS and OS in gastric cancer patients receiving adjuvant therapy following curative resection. Its reliance on routinely available biochemical parameters makes ALBI an easily obtainable prognostic measure. Validation of our findings in prospective, multicenter cohorts, along with the integration of the ALBI score with inflammatory, nutritional, and other prognostic indices, may enable more refined risk stratification during the adjuvant period. In conclusion, the ALBI score is a potential biomarker that may support postoperative risk stratification in gastric cancer patients receiving adjuvant therapy. ALBI should be interpreted as a prognostic marker rather than a tool to guide specific clinical management decisions.

## Figures and Tables

**Table 1:** Baseline clinicopathological characteristics of the study population. Values are presented as n (%).

	ALBI Grade 1 (n = 95)	ALBI Grade 2 (n = 73)	*p*
Age			0.014
<65 years	71 (74.7)	42 (57.5)	
≥65 years	24 (25.3)	31 (42.5)	
Gender			0.016
Female	38 (40)	17 (23.3)	
Male	57 (60)	56 (76.7)	
Smoking status			0.255
Smoker	37 (38.9)	33 (45.2)	
Non-smoker	58 (61.1)	40 (54.8)	
Dissection Type			0.274
D1	38 (40)	25 (34.2)	
D2	57 (60)	48 (65.8)	
Resection Type			0.126
R0	64 (67.4)	46 (63)	
R1	27 (28.4)	27 (37)	
R2	4 (4.2)	0 (0)	
Postoperative Pathological Stage			0.843
1	10 (10.5)	6 (8.2)	
2	31 (32.6)	26 (35.6)	
3	54 (56.8)	41 (56.2)	
LVI			0.395
Absent	27 (28.4)	23 (31.5)	
Present	68 (71.6)	50 (68.5)	
Grade			0.872
1	24 (25.3)	16 (21.9)	
2	32 (33.7)	25 (34.2)	
3	39 (41.1)	32 (43.8)	
PNI			0.118
Absent	34 (35.8)	19 (26)	
Present	61 (64.2)	54 (74)	
BMI			0.524
<18.5	7 (7.4)	9 (12.3)	
18.5–24.9	50 (52.6)	38 (52.1)	
≥25	38 (40)	26 (35.6)	
NLR			0.559
Low	43 (45.3)	33 (45.2)	
High	52 (54.7)	40 (54.8)	
ECOG PS			0.146
0	59 (62.1)	39 (53.4)	
1	36 (37.9)	34 (46.6)	

Abbreviations: ALBI = Albumin–Bilirubin; LVI = Lymphovascular invasion; PNI = Perineural invasion; BMI = Body Mass İndex; NLR = neutrophil-to-lymphocyte ratio; ECOG = Eastern Cooperative Oncology Group; PS = Performance status.

**Table 2:** Kaplan–Meier estimated 1-, 3-, and 5-year overall survival (OS) and recurrence-free survival (RFS) rates according to ALBI grade.

Outcome	Time	ALBI Grade 1 (%)	95% CI	ALBI Grade 2 (%)	95% CI
OS	1 year	69	59–79	56	44–68
OS	3 years	58	48–68	38	26–50
OS	5 years	46	34–58	20	10–30
RFS	1 year	60	50–70	47	35–59
RFS	3 years	53	43–63	30	20–40
RFS	5 years	43	31–55	16	6–26

Abbreviations: OS = overall survival; RFS = recurrence-free survival; CI = confidence interval.

**Table 3:** Univariate and multivariate Cox regression analyses for Overall Survival (OS).

Variable	Univariate HR (95% CI)	Univariate *p*-Value	Multivariate HR (95% CI)	Multivariate *p*-Value
Age ≥ 65	1.592 (1.071–2.367)	0.021	1.330 (0.869–2.034)	0.189
CEA (High)	1.720 (1.169–2.567)	0.006	1.350 (0.891–2.047)	0.157
ALBI Grade (Grade 2 vs. Grade 1)	1.797 (1.217–2.653)	0.003	1.699 (1.132–2.550)	0.010
Dissection Type (D2 vs. D1)	0.953 (0.640–1.421)	0.814		
Resection Type (R1–2 vs. R0)	1.143 (0.802–1.629)	0.460		
Postoperative Surgical Stage (1,2 vs. 3)	3.064 (1.994–4.709)	0.001	3.024 (1.960–4.666)	0.010

Abbreviations: HR = hazard ratio; CI = confidence interval; CEA = carcinoembryonic antigen.

**Table 4:** Univariate and multivariate Cox regression analyses for Recurrence-Free Survival (RFS).

Variable	Univariate HR (95% CI)	Univariate *p*-Value	Multivariate HR (95% CI)	Multivariate *p*-Value
Age ≥ 65	1.477 (1.003–2.174)	0.048	1.176 (0.777–1.781)	0.444
CEA (High)	1.878 (1.284–2.747)	0.001	1.591 (1.067–2.373)	0.023
ALBI Grade (Grade 2 vs. Grade 1)	1.850 (1.269–2.698)	0.001	1.767 (1.011–4.622)	0.004
Dissection Type (D2 vs. D1)	1.056 (0.716–1.557)	0.784		
Resection Type (R1–2 vs. R0)	1.112 (0.789–1.569)	0.544		
Postoperative Surgical Stage (1,2 vs. 3)	3.116 (2.061–4.711)	0.001	3.049 (2.011–4.622)	0.01

Abbreviations: HR = Hazard ratio; CI = Confidence interval; CEA = Carcinoembryonic antigen.

## Data Availability

The data that support the findings of this study are available on request from the corresponding author. The data are not publicly available due to privacy or ethical restrictions.

## Abbreviations

The following abbreviations are used in this manuscript:

ALBI	Albumin–Bilirubin
HCC	Hepatocellular Carcinoma
OS	Overall Survival
RFS	Recurrence-Free Survival
BMI	Body Mass İndex
ECOG	Eastern Cooperative Oncology Group
LVI	Lymphovascular invasion
PNI	Perineural invasion
PS	Performance Status
NLR	Neutrophil-to-Lymphocyte Ratio
AST	Aspartate Aminotransferase
ALT	Alanine Aminotransferase
INR	International Normalized Ratio
